# Emicizumab-induced photosensitivity

**DOI:** 10.1055/s-0042-1749092

**Published:** 2022-07-21

**Authors:** Pedro Asensi Cantó, Mercedes Rodríguez Serna, Pilar Lloret Madrid, Jürgen Solís Ruiz, Ana Rosa Cid Haro, Santiago Bonanad Boix, Saturnino Haya Guaita

**Affiliations:** 1 Unidad de Hemostasia y Trombosis, Servicio de Hematología, Hospital La Fe, Valencia, Spain; 2 Servicio de Dermatología, Hospital La Fe, Valencia, Spain.

**Keywords:** hemophilia therapy, hemostasis, hemophilia A/B

## Abstract

Emicizumab constitutes a novel and effective prophylaxis for hemophilia A patients with and without inhibitors. In this case report, we describe an emicizumab-induced photosensitivity that forced permanent sun-exposure suppression. To the best of our knowledge, this side effect had not been communicated until present.

A 35 year-old-man diagnosed with hemophilia A and inhibitors started prophylaxis with emicizumab. Six days after the first loading dose of 3 mg/kg (195 mg), he was exposed to the sun for 45 minutes without photoprotection, and 3 hours after he developed a cutaneous erythema in photoexposed areas ( Fig. 1A and B ). Mucosa was not involved. With the exception of emicizumab, the patient had not used any new topic or systemic photosensitizing agent. A phototest ( Fig. 1C ) evidenced erythema and edema starting from 12.5 mJ/cm ^2^ of ultraviolet B and an aberrant response to ultraviolet A starting from 7.5 J/cm ^2^ , coherent with photosensitivity. Autoimmunity blood tests, including antinuclear antibodies immunofluorescence assay, rheumatoid factor, and erythrocyte sedimentation rate, they did not show any significant alteration.

**Fig. 1:**
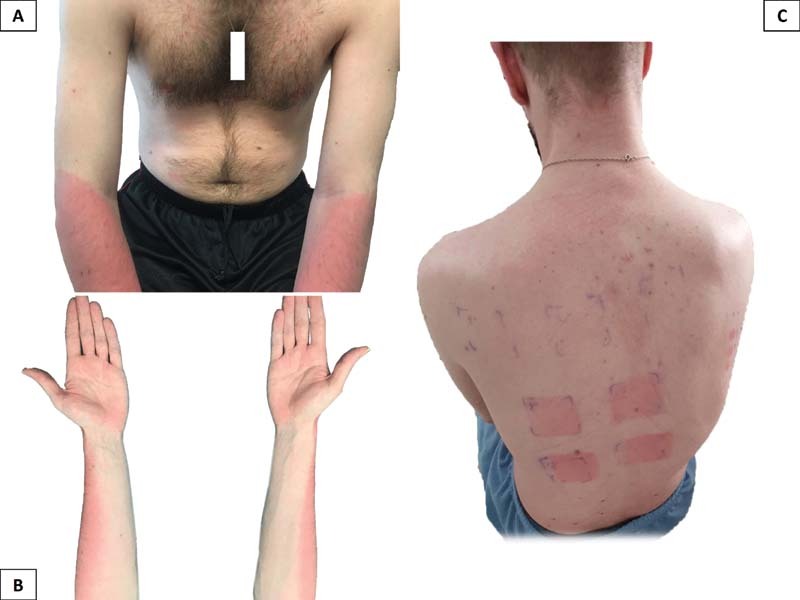
( **A, B** ) Appearance of cutaneous erythema in photoexposed areas. ( **C** ) Phototest revealing erythema and edema.

Extended-spectrum photoprotection with a sun protection factor 50+ and avoidance of prolonged sunlight exposure permitted continuation of emicizumab and the regression of lesions in 2 weeks.

Emicizumab constitutes a novel and effective prophylaxis for hemophilia A patients with and without inhibitors. This report describes the first case of photosensitivity associated with emicizumab therapy. Photoprotection is only partially effective to prevent skin lesions produced by ultraviolet radiation. Therefore, sunlight avoidance, especially in maximal ultraviolet irradiation hours, is still necessary. Prolonged sun-exposure suppression may be a quality of life–deteriorating side effect.

